# Immunomodulatory effect of the combined use of Vetosporin Zh probiotic and Gumi-malysh biologically active additive

**DOI:** 10.14202/vetworld.2021.1915-1921

**Published:** 2021-07-26

**Authors:** Alfia V. Andreeva, Aigul Z. Khakimova, Alexander I. Ivanov, Oksana N. Nikolaeva, Oleg M. Altynbekov

**Affiliations:** Department of Infectious Diseases, Zoohygiene and Veterinary Sanitary Inspection, Federal State Budgetary Educational Establishment of Higher Education “Bashkir State Agrarian University,” Ufa, Russia

**Keywords:** calves, immunomodulator, normosil, probiotic, Vetosporin Zh

## Abstract

**Background and Aim:**

Various means and methods, including probiotics and biologically active additives, have been developed and proposed for production to increase the immunobiological reactivity of the body, regardless of the etiology of its decrease. This study aimed to find out the immune status of calves during the preweaning period in association with Vetosporin Zh, Normosil, and Gumi-malysh.

**Materials and Methods:**

The research object was 30-day-old calves of black-and-white Holstein breed. The calves were divided into four groups of 20 heads each. The calves of the first, second, and third experimental groups were treated with Normosil probiotic, Vetosporin Zh probiotic, and Vetosporin Zh probiotic in combination with Gumi-malysh, respectively. The calves in the first, second, and third experimental groups were treated with Normosil probiotic, Vetosporin Zh probiotic, and Vetosporin Zh probiotic in combination with Gumi-malysh, respectively.

**Results:**

On days 10 and 21 of the experiment, animal blood was collected to determine the content of total protein, protein fractions, immunoglobulins, T and B lymphocytes, phagocytic activity and a phagocytic number of neutrophils, and circulating immune complexes (CIC). The combined use of Vetosporin Zh probiotic (dose, 20 mL) with Gumi-malysh (dose, 30 mL) per animal for 30 days in 1-month-old calves contributes to the increase in the number of T lymphocytes, B lymphocytes, and immunoglobulin A (IgA) and immunoglobulin G (IgG) levels by 2.9%, 3.8%, and 0.96 and 2 g/L, respectively, while reducing the immunoglobulin M (IgM) level; an increase in the phagocytic activity of blood neutrophils and the phagocytic number by 7% and 1.8%, respectively, as well as a decrease in the CIC level with similar indicators in calves that were not treated with the agents.

**Conclusion:**

The method used in the current study helps increase the number of T and B lymphocytes, increase IgA and IgG levels while reducing IgM levels, and increase the phagocytic activity and a phagocytic number of blood neutrophils, as well as decrease the CIC level.

## Introduction

In recent years, consumers are increasingly thinking not only about the quality of food but also about the food and environmental safety of animal products. New biologically active preparations — probiotics — are most effective in the complex solution of these problems. In modern animal husbandry, probiotic feed additives are used not only to improve digestion but also to increase production efficiency by reducing the pathogen prevalence and the environmental impact of farm animals on the environment. Thus, probiotic preparations become a safe and natural strategy for raising young farm animals due to the ban on antibiotics as growth-promoting factors [1-4].

Probiotics are a live microbial feed additive that positively affects the host animal, improving its microbial balance [5-7]. As a rule, probiotics are developed based on bifidobacteria; lactobacillus and *Escherichia*; and nonpathogenic bacilli; streptococci, and enterococci isolated from the intestines of healthy people and animals or food products. The microorganisms that are part of probiotics are not pathogenic, not toxic, can be found in sufficient quantities, and remain viable when passing through the gastrointestinal tract and stored [8].

Numerous studies have found that the use of probiotics as growth stimulators and therapeutic and prophylactic agents for young farm animals is very encouraging, and the application scope in practical animal husbandry is unlimited [9-11].

Jørgensen *et al*. [12] studied the effect of *Bacillus*-based probiotic supplements (a mixture of spray-dried spore-forming *Bacillus licheniformis* [DSM 5749] and *Bacillus subtilis* [DSM 5750]) on the growth indicators and the absorption of nutrients in piglets from weaning to fattening. They found that the probiotic significantly improved the growth rate during the growing period (70-120 days) by 11.2%.

Joysowal *et al*. [4] performed an experiment to evaluate the effect of *Pediococcus acidilactici* FT28 probiotic, which was isolated from the pig intestines. This study found that probiotics based on *Lactobacillus acidophilus* NCDC 15 and *P. acidilactici* FT28 are useful for raising pigs in terms of growth, feed conversion efficiency, and nutrient absorption. Probiotic preparations improved the biochemical blood profile, meat quality, and intestinal morphology in growing pigs.

Ventsova and Safonov [13] conducted a study on the stimulating effect of probiotics in calves with growth retardation. The calves were treated with *Bacillus amyloliquefaciens* C-1 (experimental group *Ba*) and *B. subtilis* (experimental group *Bs*), the control group was intact for 30 days. The calves in groups *Ba* and *Bs* showed increased body weight gain, feed intake, and *GH/IGF-1* levels, as well as a more effective feed conversion rate, compared with the control group (p<0.05). Moreover, the number of bacteria contributing to energy production, including *Proteobacteria*, *Rhodospirillaceae*, *Campylobacterales*, and *Butyricimonas*, was higher compared with the control group (p<0.05); the number of pathogens, including anaeroplasma and acholeplasma, decreased (p<0.05) in groups *Bs* and *Ba*. Furthermore, Akkermansia, involved in the immune response of the intestinal mucosa, was increased in group *Bs* but did not show an obvious difference in group *Ba*.

Many researchers [7,14-17] described probiotics as an effective natural strategy for improving animal health and productivity. In animals, probiotics can perform immunomodulatory functions even in low doses, proving a close relationship between the immune status of the body and the microflora population in the gastrointestinal tract.

The study conducted by Xu *et al*. [18] showed that a probiotic feed supplement containing *Lactobacillus casei* Zhang, *Lactobacillus plantarum* P-8, and *Bifidobacterium animalis* subsp*. lactis* V9 improved animal health when used in dogs. In particular, probiotics increased the level of serum immunoglobulin G (IgG; p<0.001), interferon-alpha (p<0.05), and secretory immunoglobulin A (IgA) in fecal matter (p<0.001), while reducing tumor necrosis-alpha (p<0.05). Moreover, probiotics significantly increased the number of beneficial bacteria (including some species of *Lactobacillus* and *Faecalibacterium prausnitzii*) and reduced the number of potentially harmful bacteria (including *Escherichia coli* and *Sutterella stercoralis*), and older dogs showed the strongest response to probiotics. The relative abundance of some of these species correlated with certain immune factors and physiological parameters [18].

Some effects of probiotic modulation include cytokine production by epithelial cells, increased mucin secretion, increased phagocytosis activity and activation of T cells and natural killer T cells, stimulation of IgA production, and decreased T cell proliferation [1,19-24]. Noteworthy are the studies conducted by Uyeno *et al*. on Holstein calves treated with a probiotic consisting of *L. plantarum*, *Enterococcus faecium*, and *Clostridium butyricum*. Scientists found that the number of CD^3+^ T cells; CD^4+^, CD8^+^, and WC^1+^, gd T cells; and CD^14+^, CD^21+^, and CD2^82+^ (TLR2) cells was significantly increased in healthy and experimental calves on day 7 [25].

However, the literature data analyzed are still ambiguous in their interpretations and are scattered. The general mechanisms and specific features of the various probiotic bacteria that interact with the intestinal microbiota and the immune system are incredibly complicated and not yet fully understood, which significantly complicates the use of probiotic agents in veterinary medicine.

Many scientific papers around the world are devoted to studying the effect of various probiotics on the intestinal microbiocenosis of farm animals, animal growth and development, as well as on some other indicators [4,6,10,11]. However, insufficient attention has been paid to studying the immunomodulatory effect of probiotics, which is relevant today.

For the first time, the authors conducted comprehensive studies of the dynamics of morphological parameters of blood, protein spectrum, factors of immunological reactivity of calves during the preweaning period when using the combination of “Vetosporin-Zh” and “Gumi-malysh”, thereby an effective method for correcting the immune status of animals was found.

This study aimed to find out the immune status of calves during the preweaning period against the background of Vetosporin Zh, Normosil, and Gumi-malysh.

## Materials and Methods

### Ethical approval

The study was conducted in accordance with the ethical principles approved by the Animal Experiments Ethics Committee, Federal State Budgetary Educational Institution of Higher Professional Education “Bashkir State Agrarian University” (Protocol No. 8 of 28.03.2019).

### Study period and location

The study was carried out from September 2017 to March 2020 under the conditions of Alekseevsky state farm, Ufa district, the Republic of Bashkortostan.

### Animals and donors

The research object was 30-day-old calves of black-and-white breed Holstein. The research animals were selected according to the analog principle and were in the same feeding and maintenance conditions.

The work used:

1. Vetosporin Zh — a probiotic agent containing the biomass of *B. subtilis* 12B and *B. subtilis* 11B spore bacteria in the culture medium, producing ­biologically active compounds that improve the breakdown of feed nutrients, increasing their availability to the animal body, contributing to metabolism improvement, and preventing the development of opportunistic pathogenic microflora. Vetosporin Zh promotes the synthesis of antimicrobial substances (Subtilin, Ericin S, Mersacidin, Surfactin, Bacilysin, Bacitracin, and Difficidin) and secretes ribosomally synthesized peptides (bacteriocins: types A and B lantibiotics), nonribosomal synthesized peptides (lipopeptides), and nonpeptides (polyketides, amino sugars, and phospholipids). The total number of viable cells (*B. subtilis* 12B and *B. subtilis* 11B) in 1 mL of Vetosporin Zh probiotic is not <1×108 colony-forming units (BashInkom Research and Innovation Company LLC, Ufa, Republic of Bashkortostan, Russia).
2. Normosil — a new-generation probiotic containing a mixture of live cultures, including strains of lactic acid bacteria and enterococci (*Lactobacillus brevis*, *L. plantarum*, *L. acidophilus*, *E. faecium*, and inactivated yeast saccharomycetes, the total titer is not <1×108 c/mL). This probiotic ensures metabolism recovery and releases several vital amino acids, enzymes, vitamins (group B, C, folic acid, and so on), and also improves Hb level; increases the absorption of calcium, iron, and other micro and macronutrients; and increases the body’s resistance to infections. It promotes interferon and lysozyme production (BashInkom Research and Innovation Company LLC).
3. Gumi-malysh — a biologically active additive and is a suspension containing a fine break of brown coal. The active constituent is humic acid. It contains calcium, phosphorus, and trace elements (BashInkom Research and Innovation Company LLC). The calves were divided into four groups of 20 heads each. The control group remained intact. The calves of the first experimental group were administered Normosil probiotic (dose, 30 mL per animal). The calves of the second experimental group were treated with Vetosporin Zh probiotic (dose, 20 mL per animal). The calves of the third experimental group were given Vetosporin Zh probiotic (dose, 20 mL) in combination with Gumi-malysh (dose, 30 mL per animal). The agents were given once a day for 21 days. The doses of the agents were determined based on the manufacturer’s recommendations.

Blood samples were collected for biochemical and immunological studies in the morning (before the main feeding) before the start of the experiment, then on days 10 and 21 of the experiment.

### Blood sampling, isolation, and processing test

The total protein amount and the protein fraction concentration in the blood serum were determined with Cobas 6000, a brand new automatic biochemical and enzyme immunoassay modular analyzer from Roche Diagnostics Deutschland GmbH (Mannheim, Germany).

The number of serum immunoglobulins A, M, and G in experimental animals was carried out with Real Best immunochemical automatic analyzer (Bashinkom, Russia). The sets used are tIgA (EIA-BEST), tIgM (EIA-BEST), and tIgG (EIA-BEST). This method is based on a two-stage *sandwich* enzyme-linked immunosorbent assay (ELISA) using monoclonal Ig-to-total antibodies. Moreover, quantitative determination of circulating immune complexes (CIC) was carried out by the ELISA technique.

### Statistical analysis

The statistical processing of the obtained data was carried out using the Biostatistics software package and Microsoft Office Excel 2013. The statistical significance between the groups in terms of quantitative characteristics was assessed using the Student’s t-test.

## Results and Discussion

In the study of the protein spectrum of the blood serum of calves, favorable changes in the metabolism in the experimental groups, which can be judged by the concentration of total protein and its fractions in the blood serum, were noted. The study of the protein spectrum of the blood serum showed that the background value of total protein ranged from 59.40±1.47 to 61.00±0.83 g/L.

**Figure-1:**
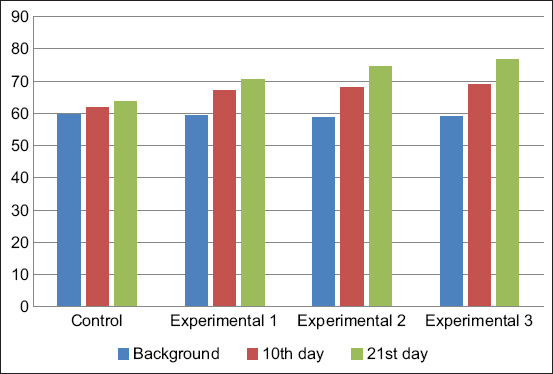
Dynamics of total protein content in blood serum, g/L.

The total serum protein content increased in calves of all groups during the experiment. However, the maximum values were achieved in the calves of the experimental groups. The highest level of total serum protein was observed in calves of the third experimental group, treated with Vetosporin Zh probiotic in combination with Gumi-malysh (Figure-1).

**Figure-2:**
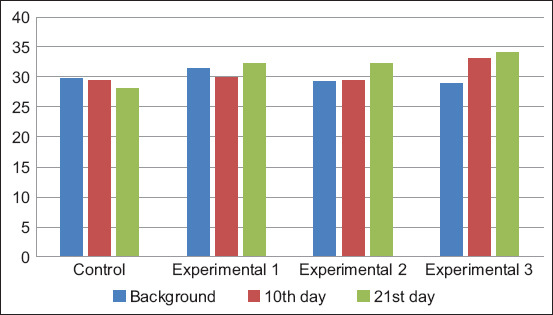
Dynamics of globulin content in blood serum, g/L.

The background value of the globulin content in the blood serum of calves ranged from 29.24±0.90 to 31.4±0.67 g/L (Figure-2). By the end of the experimental period, the maximum values of this indicator were recorded in calves of the second and third experimental groups.

The analysis of the obtained data showed that the background level of T lymphocytes (E-rosetting lymphocytes) in the blood of the calves during the preweaning period was from 59.40±3.04 to 62.40±2.20%. During the experiment, an increase in this indicator from 62.00±2.70 to 70.00±0.01% was observed in the calves of the control group. The increase in T lymphocyte content in calves of the first, second, and third experimental groups relative to the control was 4.8%, 4.8%, and 5.2%, respectively, on day 10 of the study and 1.45%, 2.8%, and 2.9%, respectively, on day 21 of the study.

The most significant increase in the level of B lymphocytes was also observed in the animals of the experimental groups. The difference in this indicator between the control and the first, second, and third experimental groups was 1.17%, 1.16%, and 2.16%, respectively, on day 10 of the study and 3.5%, 3.6%, and 3.8%, respectively, on day 21 of the study.

**Figure-3:**
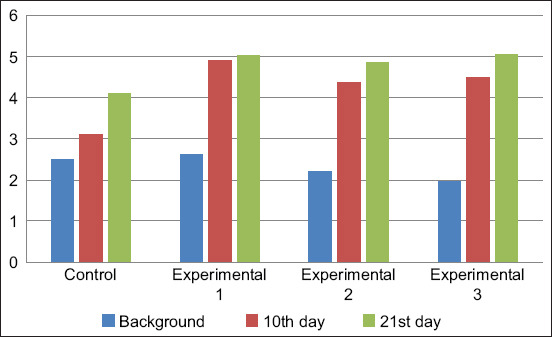
Dynamics of immunoglobulin A content in blood serum, g/L.

The study results of the immunoglobulin content in the blood serum of the calves showed that the background value of IgA was between 1.96±0.35 and 2.61±0.83 g/L (Figure-3). During the experimental period, the calves of all groups showed a tendency to increase IgA levels with the background indicator. However, the indicator of the experimental groups significantly exceeded the control values. The IgA level in the blood serum of calves of the first, second, and third experimental groups exceeded the control values by 1.18, 1.27, and 1.38 g/L on day 10 of the study, respectively, and 0.92, 0.76, and 0.96 g/L on day 21 of the study, respectively.

**Figure-4:**
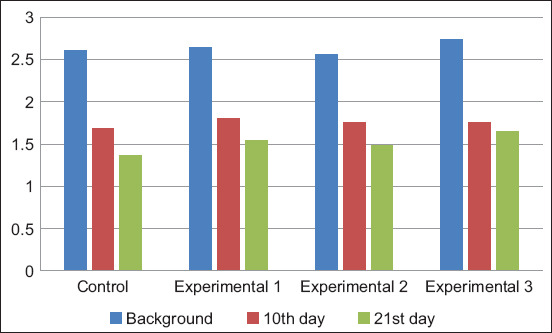
Dynamics of immunoglobulin M content in blood serum, g/L.

The background immunoglobulin M (IgM) index ranged from 2.54±0.18 to 2.88±0.12 g/L (Figure-4). Subsequently, its decrease was recorded in all groups. The dynamics of the decrease in IgM were less pronounced in the experimental groups than in the control group.

**Figure-5:**
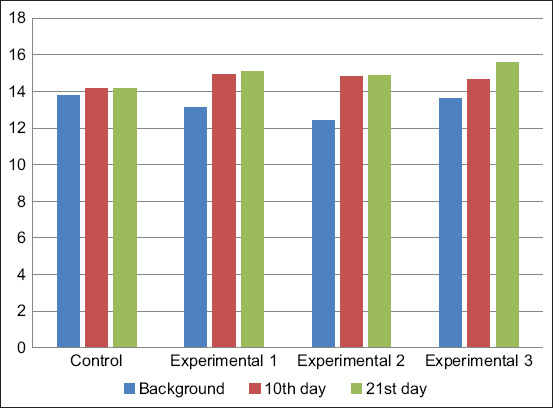
Dynamics of immunoglobulin G content in blood serum, g/L.

The background value of IgG ranged from 12.24±0.95 to 14.26±0.16 g/L (Figure-5). The IgG content in the blood serum increased by the end of the experiment in all animals. However, the maximum increase occurred in the calves of the experimental groups. The maximum values of this indicator were noted in the blood serum of the calves of the third experimental group.

**Figure-6:**
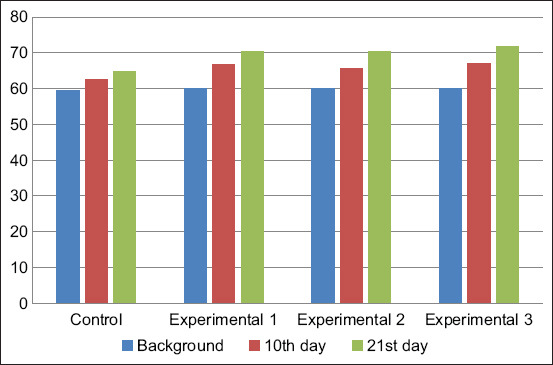
Phagocytic activity of blood neutrophils, %.

The neutrophil phagocytic activity in the animal blood at the beginning of the experimental period was 59.42±4.74–60.13±1.96% (Figure-6). This indicator reached the maximum values in the calves of the experimental groups. Thus, in the calves of the first, second, and third experimental groups, the neutrophil phagocytic activity indicator exceeded the control values by 4.2%, 3%, and 4.6%, respectively, on day 10 of the study and by 5.51%, 5.4%, and 7%, respectively, on day 21 of the study.

**Figure-7:**
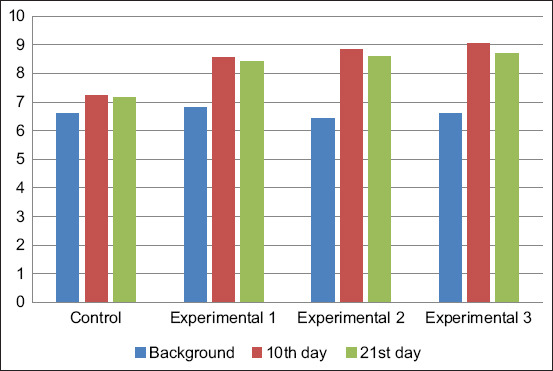
Phagocytic number of blood neutrophils, %.

The phagocytic number of blood neutrophils in calves was from 6.60±0.24 to 6.8±0.67% at the beginning of the experiment (Figure-7). The most significant increase in this indicator was observed in the animal blood of the experimental groups. The phagocytic number of blood neutrophils in the calves of the first, second, and third experimental groups exceeded that of the control group on day 10 of the study by 1.3%, 1.6%, and 1.8%, respectively. However, a slight decrease in this indicator was observed in all experimental groups on day 21 of the study.

**Figure-8:**
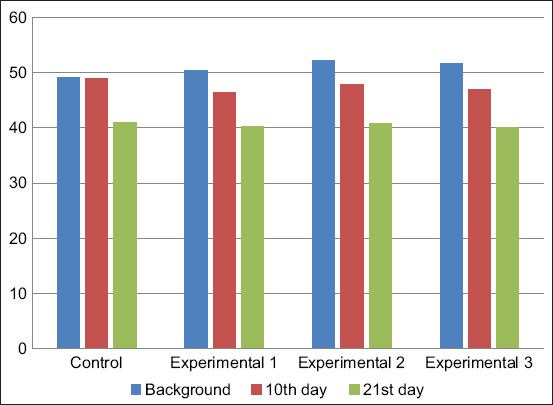
Dynamics of circulating immune complexes content, AU.

The use of the agents showed a tendency to decrease CIC in the blood serum within the physiological norm during the entire study period (Figure-8). The lowest value of the CIC content on day 21 of the study was registered in the blood serum of the calves of the third experimental group. A low level of CIC in the blood serum of animals indicates the absence of inflammatory diseases, including infectious etiology, which could cause pathology development. Moreover, Dar *et al*. [8] found that the use of probiotics and prebiotics in 15-day-old crossbred calves increases the bactericidal, lysozyme, and complementary activity of blood serum and reduces the CIC content in cows and calves.

de LeBlanc *et al*. [26], in their studies conducted on laboratory animals, found that fermented dairy products containing probiotic bacteria contribute to IgA increase. It has also been experimentally proven that long-term use of probiotics increases macrophage phagocytic activity, which, in addition to neutrophils, is involved in the implementation of innate immunity mechanisms.

Xu *et al*. [18], scientists from China, conducted experiments on dogs in a canine training ground and found that probiotics have a pronounced immunomodulatory effect. In dogs treated with probiotic agents, a significant increase was noted in serum IgG and secretory (fecal) IgA levels during the period of agent use and 15 days after probiotic discontinuation, compared with both the corresponding control and background levels.

Dowarah *et al*. [1], Indian scientists, evaluated the indicators of humoral immunity after the probiotic use in their studies conducted on piglets in the post-weaning period. The data analysis showed that the serum level of IgM concentration increased in the groups of piglets with a probiotic-based diet.

Ventsova and Safonov [13] conducted studies on the effect of probiotic agents based on *B. amyloliquefaciens* and *B. subtilis* on improving the growth and development of calves. The experiments found that the levels of serum IgA, IgM, and IgG were increased compared with similar indicators in the control group, although not significantly, in calves treated with probiotics.

In the current study, the mechanism of the complex effect of a probiotic with a prebiotic is indirectly presented. Thus, the use of probiotics was previously shown to help reduce the antigenic load due to the bacillus antagonistic activity. However, the current study established the integrated effect of probiotics and prebiotics on phagocytic activity growth, that is, increased absorption of pathogenic and opportunistic microorganisms, which may indicate that the target of their influence is cellular and not humoral arm. Moreover, this also explains the effect of CIC lowering.

## Conclusion

Thus, the study results enabled the conclusion that the use of Normosil and Vetosporin Zh probiotics, as well as the combined use of Vetosporin Zh with Gumi-malysh, has a beneficial effect on the immunobiological status of the calves during the preweaning period. The combined use of Vetosporin Zh probiotic (dose, 20 mL) with Gumi-malysh (dose, 30 mL per animal) for 30 days in 1-month-old calves contributes to:

- An increase in the number of T and B lymphocytes by 2.9% and 3.8%, respectively
- An increase in IgA and IgG levels by 0.96 and 2 g/L, respectively, while reducing the IgM level
- An increase in the phagocytic activity of blood neutrophils and the phagocytic number by 7% and 1.8%, respectively, as well as a decrease in CIC level with similar indicators in calves that were not treated with the agents.

## Authors’ Contributions

AVA: Developed the study, supervised the work, evaluated and analyzed the data, compiled and edited the manuscript. AZK: Conducted the experiment, delivered the biological material to the laboratory. ONN: Conducted laboratory tests. AII: Performed statistical analysis of the data obtained. OMA: Collected and processed the literature data on the research topic. All authors analyzed the data and approved the final version of the manuscript.
